# Comparative Photopic and Mesopic Visual Performance of Enhanced Monofocal Versus Non-Diffractive Extended Depth-of-Focus Intraocular Lenses

**DOI:** 10.3390/jcm15041368

**Published:** 2026-02-09

**Authors:** Inas Baoud Ould Haddi, Vanesa Blázquez-Sánchez, Dayan Flores-Cervantes, Emilio Dorronzoro-Ramirez, Nuria Garzón, Cristina Bonnin-Arias

**Affiliations:** 1 Optometry and Vision Department, Faculty of Optics and Optometry, Complutense University of Madrid, 28037 Madrid, Spain; ibaoud@ucm.es (I.B.O.H.); vblazquez@ucm.es (V.B.-S.); cbonnina@ucm.es (C.B.-A.); 2 Ophthalmology Department, Sanitas La Moraleja Hospital, 28050 Madrid, Spain; mdflores@sanitas.es (D.F.-C.); edorronzoro@sanitas.es (E.D.-R.)

**Keywords:** extended depth-of-focus, enhance monofocal, intraocular lens, mesopic visual performance

## Abstract

**Background/Objectives**: Enhanced monofocal and non-diffractive extended depth-of-focus (EDoF) intraocular lenses (IOLs) aim to improve intermediate vision while maintaining contrast sensitivity. However, direct comparative evidence under both photopic and mesopic conditions remains limited. This study prospectively compared the visual performance of two enhanced monofocal IOLs (Tecnis^®^ Eyhance^™^ and ISOPure^®^) and one non-diffractive EDoF IOL (AcrySof^®^ IQ Vivity^™^). **Methods**: Sixty patients undergoing bilateral cataract surgery were implanted with one of three IOL groups (n = 20 each). Patients were assigned to one of three IOL groups based on preoperative consultation and clinical indications. One month postoperatively, monocular corrected distance (CDVA), distance-corrected intermediate (DCIVA), and near visual acuity (DCNVA) were measured under photopic and mesopic conditions. Photopic defocus curves, contrast sensitivity under photopic and mesopic conditions and correlation between pupil diameter and visual acuities were also assessed. **Results**: Baseline characteristics were comparable among groups. Under photopic conditions, Vivity^™^ achieved significantly better UDVA, DCIVA and DCNVA than both Eyhance^™^ and ISOPure^®^. Under mesopic conditions, distance acuity did not differ significantly, but Vivity™ showed superior DCIVA and DCNVA. Defocus curves demonstrated a broader functional range of vision with Vivity™, while Eyhance^™^ and ISOPure^®^ showed nearly overlapping profiles. Contrast sensitivity was similar among all IOLs under both lighting conditions, with no statistically significant differences. **Conclusions**: The non-diffractive EDoF AcrySof^®^ IQ Vivity^™^ provided a wider and more functional depth-of-focus than the enhanced monofocal lenses evaluated, without compromising contrast sensitivity. Eyhance^™^ and ISOPure^®^ offered comparable performance, with good distance vision and modest depth-of-focus extension. All three IOLs maintained CS levels comparable to those typically reported for standard monofocal IOLs under both photopic and mesopic illumination, indicating no clinically relevant contrast penalty.

## 1. Introduction

Cataract surgery has evolved from a procedure focused solely on restoring visual clarity to one aimed at achieving a high degree of postoperative spectacle independence. Contemporary patients increasingly expect high-quality vision across a broad range of distances, driven by modern visual demands and the omnipresence of digital devices. To address these expectations, successive generations of intraocular lens (IOL) technologies have been developed [1].

Early multifocal IOLs, initially bifocal designs, provided functional distance and near vision but were frequently associated with optical side effects, particularly halos, glare, and reduced performance under mesopic conditions due to their intrinsic pupil dependence [2,3]. The advent of trifocal IOLs expanded functional vision to include the intermediate range, offering a more complete refractive solution and reducing spectacle dependence [4]. However, despite improved range of vision, many patients continued to experience dysphotopsias inherent to diffractive optics [5].

In response, a new class of lenses, extended depth-of-focus (EDoF) IOLs, was introduced to provide smoother through-focus performance with fewer photic phenomena. These designs employ optical strategies that generate a continuous elongated focal zone, enhancing vision from distance to intermediate while aiming to preserve contrast sensitivity and reduce unwanted visual disturbances [6,7]. Nevertheless, most EDoF IOLs still provide limited near vision, and many patients continue to require reading glasses for fine-print tasks [3].

There is growing clinical interest in monofocal intraocular lenses designed to enhance intermediate vision, yet their actual performance relative to established extended depth-of-focus technologies remains insufficiently characterized. The terminology itself has contributed to confusion: lenses marketed as EDoF are generally understood to rely on modified refractive or non-diffractive wavefront-shaping profiles, whereas enhanced monofocal or advanced monofocal lenses are intended to preserve the visual quality of a monofocal design while offering a modest extension of functional depth-of-focus. However, the true extent to which these lenses differ in real-world performance is still uncertain [8].

Although several publications have reported outcomes for each lens individually, direct, controlled, head-to-head comparisons are scarce, and the existing studies vary widely in visual endpoints, testing distances, and illumination conditions. This heterogeneity limits the ability to draw firm conclusions about the relative efficacy of intermediate-boosting monofocal lenses versus EDoF designs, defined as those meeting ANSI criteria. Moreover, evidence under mesopic conditions is particularly limited, despite the clinical relevance of low-illumination performance for tasks such as night driving. Consequently, it remains unclear whether the optical strategies used by enhanced monofocal lenses achieve a clinically meaningful extension of depth-of-focus without adversely affecting contrast sensitivity or low-light visual quality.

Given the rapid and widespread adoption of these lenses in routine cataract surgery, surgeons need robust comparative data to understand how much functional intermediate and near vision can realistically be expected from each design and to determine whether any observed differences are clinically significant.

To address these knowledge gaps, we conducted a prospective, controlled comparison of three depth-of-focus–enhancing IOLs, two enhanced monofocal lenses (Tecnis^®^ Eyhance^™^ (Johnson & Johnson Vision, Jacksonville, FL, USA), and ISOPure^®^ (BVI medical, Liège, Belgium)), and one EDoF lens (AcrySof^®^ IQ Vivity^™^ (Alcon Laboratories, Fort Worth, TX, USA)), marketed as a non-diffractive EDoF IOL, and has been reported to meet ANSI Z80.35 criteria according to manufacturer documentation, assessing photopic and mesopic visual acuity, defocus curves, and contrast sensitivity under standardized testing conditions.

## 2. Materials and Methods

This prospective, non-randomized comparative study was conducted at Hospital Universitario Sanitas La Moraleja (Madrid, Spain) between November 2021 and December 2022. The protocol was approved by the Ethics and Clinical Research Committee of Hospital Clínico San Carlos (reference number 21/642-O_P) and adhered to the principles of the Declaration of Helsinki and Good Clinical Practice. Written informed consent was obtained from all participants.

The sample size was calculated using the Granmo calculator (version 7.12, Institut Municipal d’Investigació Mèdica, Barcelona, Spain). The primary variable used for the calculation was monocular intermediate visual acuity at 66 cm under photopic conditions. To detect a clinically relevant difference of 0.1 logMAR between IOL groups, assuming a standard deviation of 0.1 logMAR, obtained from an internal pilot dataset collected in our center, using a two-sided alpha of 0.05 and 80% power, a minimum of 18 eyes per group was required. A total of 20 eyes per group (20 patients) were finally included, meeting and exceeding the required sample size.

Eligible participants were adults (≥50 years) of either sex, diagnosed with bilateral cataracts and presenting with clinically relevant visual symptoms and/or decreased visual acuity. Patients were excluded if they had ocular comorbidities such as severe dry eye, active or past uveitis, retinal disease, or glaucoma. Patients were also excluded if they presented with corneal pathology or irregular astigmatism. Regular corneal astigmatism > 1.00 D was also considered an exclusion criterion. Additional exclusion criteria included uncontrolled systemic conditions that could interfere with the study outcomes and any ocular surgery within the six months prior to enrolment.

The choice of lens was made during the preoperative consultation, based on the patient’s visual needs, lifestyle preferences, and the surgeon’s recommendation.

### 2.1. Intraocular Lenses

The intraocular lenses implanted in this study presented the following technical specifications:

The Tecnis^®^ Eyhance^™^ (Johnson & Johnson Vision, USA) is an enhanced monofocal intraocular lens designed to increase depth-of-focus while preserving the optical quality of conventional monofocal lenses. It employs a continuous refractive optic without rings, with a power gradient increasing from the periphery to the center, creating a high-order aspheric anterior surface that enhances intermediate vision without affecting distance acuity [9]. The lens is designed to provide −0.27 µm of primary spherical aberration for a 6 mm pupil [10]. It is a single-piece hydrophobic acrylic IOL with an optical diameter of 6.00 mm and a total diameter of 13.00 mm. It includes a UV filter. Its dioptric range spans from +5.0 D to +34.0 D in 0.5 D increments.

The ISOPure^®^ (BVI medical, Belgium) is a single-piece IOL made of hydrophobic acrylic, with an optical diameter of 5.75 mm and a total diameter of 11.00 mm. It is a fully refractive lens that incorporates a proprietary isofocal optical design. This architecture is based on a continuous modulation of optical power across the anterior and posterior surfaces, with a central zone of increased curvature that transitions smoothly toward the periphery. Through this optical strategy, the lens achieves a modest extension of depth-of-focus compared with conventional monofocal IOLs, while preserving robust distance visual performance [10,11]. It incorporates ultraviolet and blue light filters and has a negative asphericity of −0.11. The available power range extends from +10.0 D to +30.0 D in 0.5 D increments, and from +31.0 D to +35.0 D in 1.0 D increments.

The AcrySof^®^ IQ Vivity^™^ (Alcon, USA) is an FDA-approved, non-diffractive EDoF IOL. It meets industry standards, including those from the American National Standards Institute (ANSI) for EDOF lenses, offering improved intermediate/near vision compared to standard monofocal lenses while maintaining good distance vision. The IOL features an aspheric anterior surface with a centrally located modification based on a toroidal geometry, described by the manufacturer as a non-diffractive wavefront-shaping mechanism (X-WAVE™ technology). This design incorporates two distinct transition elements within the anterior optic. The first is a subtle central elevation that elongates the propagating wavefront, thereby creating a continuous extension of the focal region. The second is a controlled variation in curvature that redistributes the extended focus along the optical axis, ensuring that the available light energy remains functionally effective. Without this axial redistribution, the elongated focus would be symmetrically distributed anterior and posterior to the retina, thereby reducing its clinical utility. By shifting the focal distribution toward the myopic side, the design concentrates usable optical energy within a single extended focal zone. Together, these transition elements act concurrently to provide a smooth and continuous range of vision without diffractive light splitting. The lens incorporates a negative spherical aberration with a nominal value of approximately −0.20 μm [12]. It is a single-piece IOL composed of hydrophobic acrylic copolymer, with an optical diameter of 6.00 mm and a total diameter of 13.00 mm. It features UV and blue light filters, and a power range of +10.0 D to +30.0 D in 0.5 D increments.

### 2.2. Protocol Evaluation

Preoperative assessment included uncorrected and corrected distance visual acuity (UDVA, CDVA), autorefraction with the Zeiss Visuref^®^ 150 (Carl Zeiss Meditec, Jena, Germany), intraocular pressure measured with the CT-80^®^ non-contact tonometer (Topcon, Tokyo, Japan), endothelial cell count with the SP-3000P^®^ specular microscope (Topcon, Japan), corneal tomography with the Pentacam^®^ (Oculus, Wetzlar, Germany), and macular evaluation with optical coherence tomography using the Dri OCT Triton^®^ (Topcon, Japan). IOL power and predicted residual refraction were calculated with the Barrett Total K^®^ formula, integrated in the IOL Master 700^®^ optical biometer (Carl Zeiss Meditec, Jena, Germany). The selected IOL corresponded to the first negative predicted residual value.

One month after surgery, visual acuity was assessed under photopic (≥85 cd/m^2^) and mesopic (3.5 cd/m^2^) conditions. The evaluation included corrected distance visual acuity (CDVA) at 4 m, distance-corrected intermediate visual acuity (DCIVA) at 66 cm, and distance-corrected near visual acuity (DCNVA) at 40 cm, all measured with 100% contrast ETDRS charts (Precision Vision, Woodstock, IL, USA). For monocular analyses, only the right eye (pre-specified as the first operated) was included to avoid within-subject clustering. Monocular defocus curves were obtained under photopic conditions at 4 m using 100% contrast ETDRS charts, introducing spherical trial lenses from −3.00 D to +1.00 D in 0.50 D increments over the best distance correction. Contrast sensitivity was assessed with the CSV-1000 (VectorVision, Greenville, OH, USA) at 2.50 m under both photopic and mesopic illumination.

Photopic pupil diameter was recorded with the Pentacam^®^ (Oculus, Wetzlar, Germany), and Mesopic pupil diameter was recorded with the Power Refractor II (Plusoptix, Nürnberg, Germany). All visual assessments were performed following a standardized testing sequence. Photopic measurements were always conducted first, after which patients were allowed a 5 min adaptation period before mesopic testing. Mesopic visual acuity assessments were then performed under controlled low-luminance conditions. Pupil diameter was measured under illumination levels matching those used for the corresponding visual acuity testing conditions.

### 2.3. Surgical Procedure

All cataract surgeries were performed by the same experienced surgeon (EDR) under topical anesthesia. An anterior capsulotomy (5.2 mm diameter) and nuclear fragmentation (three 7 mm radial cuts) were created with the Victus^®^ femtosecond laser (Bausch & Lomb GmbH, Berlin, Germany). A 2.2 mm clear corneal incision was made superotemporally in the right eyes and superonasally in the left eyes. Phacoemulsification was performed with the Stellaris Elite^®^ microsurgical platform (Bausch & Lomb GmbH, Berlin, Germany), and the allocated intraocular lens was implanted in the capsular bag using a single-use injector. The CALLISTO Eye^®^ system (Carl Zeiss, Jena, Germany) provided intraoperative guidance. All patients underwent second-eye surgery within 7 days of the first procedure to ensure a consistent postoperative timeline. No intraoperative or postoperative complications were observed, and no patients were excluded from the analysis.

### 2.4. Statistical Analysis

All statistical tests were performed using the SPSS software package for Windows (version 24.0, SPSS Inc., Chicago, IL, USA). Statistical analysis was conducted to compare the outcomes of the three IOL groups. Data normality was assessed using the Shapiro–Wilk test, which indicated that most variables were not normally distributed and did not meet assumptions of homogeneity of variance. Therefore, non-parametric statistical methods were applied.

Preoperative variables were compared across groups using the Kruskal–Wallis test to confirm baseline equivalence. The same test was used to analyze postoperative outcomes, including distance, intermediate, and near visual acuity, contrast sensitivity and mesopic pupil diameter, in order to identify overall differences among the IOL groups. When statistically significant results were observed (*p* < 0.05), post hoc pairwise comparisons were performed using the Mann–Whitney U test. Bonferroni correction was applied to the three pairwise comparisons (AcrySof^®^ IQ Vivity^™^ vs. Tecnis^®^ Eyhance^™^, AcrySof^®^ IQ Vivity^™^ vs. ISOPure^®^, and Tecnis^®^ Eyhance^™^ vs. ISOPure^®^), using a Bonferroni-adjusted significance level of α = 0.0167 (0.05/3). Pairwise *p*-values are reported as raw (unadjusted) Mann–Whitney U test *p*-values and were interpreted against this Bonferroni-adjusted alpha.

Defocus-curve visual acuity data were analyzed using a linear mixed-effects model to account for repeated measures across defocus levels within each subject. IOL group, defocus level (treated as a categorical factor), and the IOL × defocus interaction were included as fixed effects, and subject was included as a random intercept. Global tests of fixed effects derived from the mixed model were used to assess overall effects of IOL group, defocus level, and their interaction.

Correlations between continuous variables, such as visual acuity measures, contrast sensitivity and mesopic pupil diameter were evaluated using Spearman’s rank correlation coefficient (ρ).

## 3. Results

A total of 60 patients were included in the study, with 20 individuals assigned to each of the three IOL groups.

Table 1 summarizes the demographic and baseline ocular characteristics. No significant differences were observed among groups in age, UDVA, CDVA, keratometry (K1, K2), axial length, IOL power, photopic and mesopic pupil diameter.

### 3.1. Postoperative Manifest Refractive Outcomes

Postoperative manifest refractive outcomes are summarized in Table 2. There were no statistically significant differences among groups in postoperative sphere, cylinder, or spherical equivalent (all *p* > 0.05). Refractive accuracy was high across all groups, with 100% of eyes within ±0.50 D of SE in the Tecnis Eyhance and AcrySof^®^ IQ Vivity™ groups and 90% in the ISOPure group; all eyes were within ±1.00 D.

### 3.2. Visual Acuity

Table 3 presents the monocular visual acuity outcomes under photopic conditions, including mean values, standard deviations, and the observed minimum–maximum ranges for each IOL group. Only right eyes were analyzed.

Under photopic conditions, statistically significant differences were observed among the three intraocular lens groups. AcrySof^®^ IQ Vivity^™^ showed significantly better UDVA compared with Tecnis^®^ Eyhance^™^ and ISOPure^®^. CDVA was significantly better with AcrySof^®^ IQ Vivity^™^ compared with ISOPure^®^, while the difference versus Tecnis^®^ Eyhance^™^ was not statistically significant. DCIVA was significantly better with AcrySof^®^ IQ Vivity^™^ compared with both Tecnis^®^ Eyhance^™^ and ISOPure^®^. For DCNVA, AcrySof^®^ IQ Vivity^™^ showed significantly better outcomes compared with both Tecnis^®^ Eyhance^™^, and ISOPure^®^. No statistically significant differences were observed between Tecnis^®^ Eyhance^™^ and ISOPure^®^ for any of the evaluated photopic visual acuity outcomes.

Table 4 presents the monocular visual acuity results obtained under mesopic conditions for each IOL group.

Under mesopic conditions, no statistically significant differences were observed among the three intraocular lens groups for uncorrected or corrected distance visual acuity (UDVA and CDVA). In contrast, statistically significant differences were found for distance-corrected intermediate and near visual acuity. AcrySof^®^ IQ Vivity^™^ showed significantly better DCIVA compared with ISOPure^®^ while no significant difference was observed versus Tecnis^®^ Eyhance™. For DCNVA, AcrySof^®^ IQ Vivity^™^ showed significantly better outcomes compared with both Tecnis^®^ Eyhance^™^ and ISOPure^®^.

### 3.3. Defocus Curve

**Figure 1:**
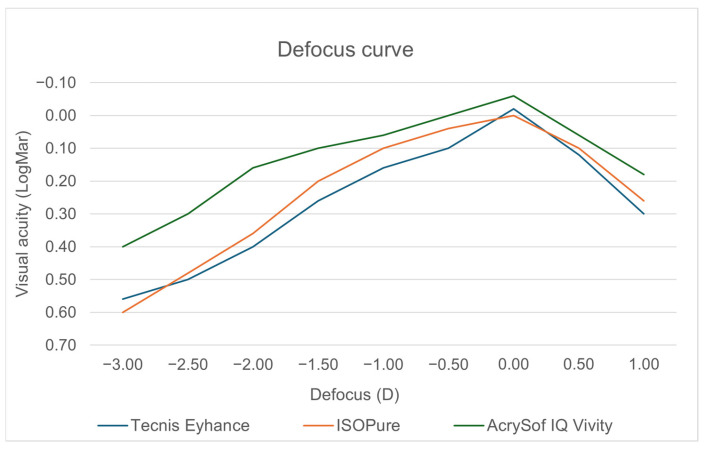
Monocular defocus curve of the right eye measured under photopic conditions. Data are presented as mean visual acuity values across defocus levels.

Using a linear mixed-effects model to account for repeated measures across defocus levels, significant effects of defocus level and IOL group were observed, as well as a significant IOL group × defocus interaction (all *p* < 0.001), indicating different through-focus visual acuity profiles among the three IOLs (Figure 1). Global mixed-model test results are provided in the Appendix A (Table A1).

Overall, the defocus curves showed distinct functional profiles across the evaluated defocus range. AcrySof^®^ IQ Vivity^™^ demonstrated a broader and more functional depth of focus compared with Tecnis^®^ Eyhance^™^ and ISOPure^®^, whose curves largely overlapped. Around the plano region, visual acuity was comparable among the three IOLs. As defocus increased toward intermediate and early near distances, Vivity™ maintained better visual acuity over a wider range, consistent with an extended depth-of-focus profile. At positive defocus levels, separation between groups was smaller, with only modest differences between curves.

### 3.4. Contrast Sensitivity

**Figure 2:**
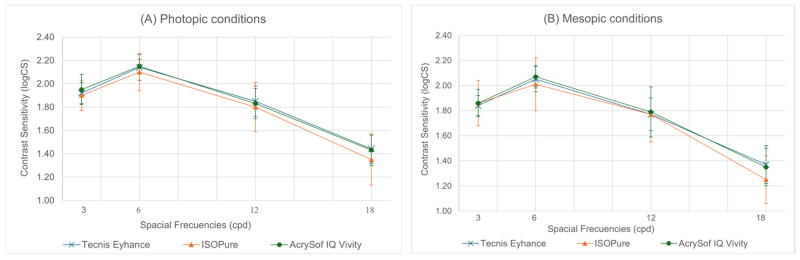
Monocular contrast sensitivity function at different spatial frequencies (3, 6, 12, 18 cpd) for the Tecnis^®^ Eyhance^™^ (blue line), ISOPure^®^ (orange line) and AcrySoft^®^ IQ Vivity^™^ (green line) under photopic (**A**) and mesopic (**B**) conditions.

Figure 2 shows the mean monocular photopic and mesopic contrast sensitivity (logCS) for the three IOL groups across all spatial frequencies, together with the corresponding statistical comparisons.

Table 5 shows the mean monocular contrast sensitivity (logCS) under mesopic conditions for the three IOL groups across all spatial frequencies, along with the corresponding statistical analyses.

Under photopic monocular conditions, all three IOLs achieved adequate contrast-sensitivity levels across the full range of spatial frequencies, with no statistically significant differences between them. Subtle performance trends were observed: Tecnis^®^ Eyhance^™^ showed slightly higher values at mid–high frequencies (12 and 18 cpd), whereas AcrySof^®^ IQ Vivity^™^ tended to perform marginally better at lower frequencies (3 and 6 cpd). ISOPure^®^ demonstrated a stable intermediate profile throughout the spectrum. These differences were not statistically significant.

Under mesopic monocular conditions, all lenses maintained functional CS levels with no statistically significant differences at any spatial frequency. At lower frequencies (3 and 6 cpd), AcrySof^®^ IQ Vivity^™^ and Tecnis^®^ Eyhance^™^ showed slightly higher values, while ISOPure^®^ performed similarly but with a modest reduction at 6 cpd. At mid and high frequencies (12 and 18 cpd), AcrySof^®^ IQ Vivity^™^ tended to show marginally higher values at 12 cpd, Tecnis^®^ Eyhance^™^, demonstrated a balanced performance, and ISOPure^®^ displayed a more noticeable decrease at 18 cpd. Overall, all lenses demonstrated comparable mesopic contrast sensitivity performance.

### 3.5. Correlation Between Pupil Diameter and Visual Acuities

**Figure 3:**
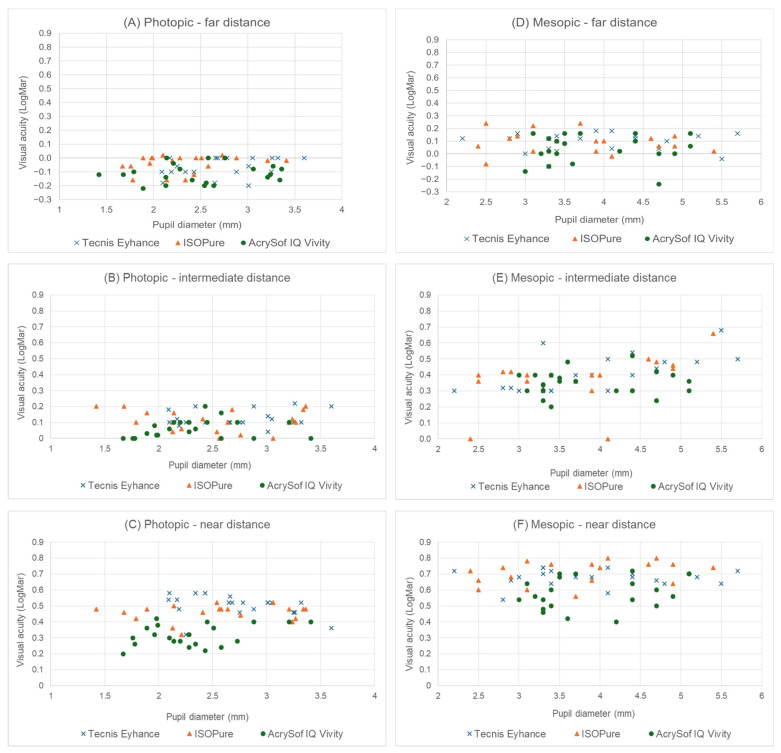
Monocular photopic and mesopic logMAR visual acuity as a function of photopic pupil diameter for three intraocular lens types: (**A**) photopic far, (**B**) photopic intermediate, (**C**) photopic near, (**D**) mesopic far, (**E**) mesopic intermediate and (**F**) mesopic near distances. Each point represents an individual eye.

No significant association was found between photopic pupil diameter and distance photopic visual acuity (logMAR) when all intraocular lenses were analyzed together (ρ = 0.15; *p*-value = 0.243) (Figure 3A–C). Subgroup analysis by intraocular lens type showed no statistically significant correlations for Tecnis^®^ Eyhance^™^ (ρ = 0.38; *p*-value = 0.100), ISOPure^®^ (ρ = 0.23; *p*-value = 0.326), or AcrySof^®^ IQ Vivity^™^ (ρ = 0.17; *p*-value = 0.479). Similarly, no significant association was observed between photopic pupil diameter and intermediate photopic visual acuity when all intraocular lenses were analyzed together (ρ = 0.20; *p*-value = 0.131). No statistically significant correlations were found for Tecnis^®^ Eyhance^™^ (ρ = 0.19; *p*-value = 0.430), ISOPure^®^ (ρ = −0.10; *p*-value = 0.672), or AcrySof^®^ IQ Vivity^™^ (ρ = 0.31; *p*-value = 0.171). No significant association was observed between photopic pupil diameter and near photopic visual acuity when all intraocular lenses were analyzed together (ρ = 0.12; *p*-value = 0.360). Subgroup analysis likewise revealed no statistically significant correlations for Tecnis^®^ Eyhance^™^ (ρ = 0.21; *p*-value = 0.372), ISOPure^®^ (ρ = 0.05; *p*-value = 0.820), or AcrySof^®^ IQ Vivity^™^ (ρ = 0.18; *p*-value = 0.444).

No significant association was found between mesopic pupil diameter and distance mesopic visual acuity (logMAR) when all intraocular lenses were analyzed together (ρ = 0.14; *p*-value = 0.280), and no statistically significant correlations were observed for Tecnis^®^ Eyhance^™^ (ρ = 0.21; *p*-value = 0.363), ISOPure^®^ (ρ = 0.18; *p*-value = 0.440), or AcrySof^®^ IQ Vivity^™^ (ρ = 0.09; *p*-value = 0.700) (Figure 3D–F).

In contrast, a significant association was observed between mesopic pupil diameter and intermediate mesopic visual acuity when all intraocular lenses were analyzed together (ρ = 0.41; *p*-value = 0.001). Subgroup analysis revealed statistically significant positive correlations for Tecnis^®^ Eyhance^™^ (ρ = 0.65; *p*-value = 0.002) and ISOPure^®^ (ρ = 0.53; *p*-value = 0.022), whereas no significant correlation was found for AcrySof^®^ IQ Vivity^™^ (ρ = 0.05; *p*-value = 0.834).

For near mesopic visual acuity, no significant association with pupil diameter was found when all intraocular lenses were analyzed together (ρ = 0.17; *p*-value = 0.200), and no statistically significant correlations were observed for Tecnis^®^ Eyhance^™^ (ρ = −0.13; *p*-value = 0.586), ISOPure^®^ (ρ = 0.37; *p*-value = 0.110), or AcrySof^®^ IQ Vivity^™^ (ρ = 0.32; *p*-value = 0.155).

## 4. Discussion

The present study provides a comparative evaluation of three depth-of-focus or enhancing IOL models (Tecnis^®^ Eyhance^™^, ISOPure^®^, and AcrySof^®^ IQ Vivity™), assessed under both photopic and mesopic conditions. Previous investigations have shown that these lenses improve functional vision across a wide range of distances, with particular benefits in the intermediate range [13,14,15]. Our analysis extends this evidence by examining their performance across varying illumination environments, which more closely reflects real-world visual demands.

Visual acuity results represent the most intuitive clinical way to evaluate the functional performance of intraocular lenses that improve depth of focus. In the present study, clear differences in visual acuity profiles were observed between the three IOLs under photopic conditions, reflecting their different optical designs.

Under photopic lighting, AcrySof^®^ IQ Vivity^™^ showed significantly better UDVA and DCIVA than both Tecnis^®^ Eyhance^™^ and ISOPure^®^, and overall better performance in CDVA and DCNVA. Postoperative manifest refractive outcomes did not differ significantly among groups (Table 2), suggesting that the observed UDVA differences are unlikely to be primarily driven by systematic differences in refractive accuracy. While differences in distance visual acuity were statistically significant, their magnitude was small, whereas the advantages observed at intermediate and near distances are more likely to be clinically relevant. AcrySof^®^ IQ Vivity^™^ showed a clinically meaningful advantage at intermediate (66 cm) and near (40 cm) distances. These findings confirm that Vivity’s non-diffractive EDoF design produces a wider functional range of vision than enhanced monofocal designs, extending beyond intermediate vision to the near range without compromising distance acuity. Notably, eyes with extreme axial lengths were uncommon in our cohort (axial length ≥ 26.0 mm: 3/60 eyes), and excluding these cases in a sensitivity analysis did not materially change the pattern of between-group differences, suggesting that our findings were not driven by axial length extremes.

When visual acuity was assessed under mesopic conditions, an overall reduction in performance was observed across all distances and intraocular lens types, consistent with the physiological effects of reduced retinal illumination, pupil dilation, and decreased contrast sensitivity. Importantly, mesopic distance visual acuity remained comparable among the three IOLs, with no statistically significant differences detected, indicating that all designs preserve stable distance vision in low-light environments, an essential requirement for visually demanding tasks such as night driving.

In contrast, differences between lenses persisted at intermediate and near distances under mesopic illumination. The non-diffractive extended depth-of-focus AcrySof^®^ IQ Vivity^™^ demonstrated significantly better performance in these ranges compared with the enhanced monofocal Tecnis^®^ Eyhance^™^ and ISOPure^®^ lenses. These findings are consistent with previous reports, including those by Savini et al. [15], who observed comparable mesopic outcomes, whereas Sabur et al. [16] reported slightly higher values, likely reflecting methodological differences in testing conditions.

From an optical standpoint, the superior intermediate and near visual acuity observed with AcrySof^®^ IQ Vivity™ can be attributed to its wavefront-shaping design, which redistributes optical energy along an extended focal zone, thereby maintaining functional retinal image quality even under low-light conditions. In contrast, enhanced monofocal designs primarily optimize distance vision with a limited extension of depth of focus, which may be more susceptible to performance degradation as illumination decreases.

The monocular defocus curve provides a more detailed representation of functional vision across different distances. In our study, when comparing the defocus curves of the three intraocular lenses evaluated in this study, all lenses demonstrated visual acuity values close to or better than 0.00 LogMAR at distance vision (0.00 D). However, the AcrySof^®^ IQ Vivity^™^ lens exhibited the best performance, achieving slightly better visual acuity values compared with Tecnis^®^ Eyhance^™^ and ISOPure^®^, suggesting a modest advantage in uncorrected distance visual quality. In the intermediate defocus range (−0.50 to −1.50 D), more pronounced differences among the lens designs were observed. The AcrySof^®^ IQ Vivity^™^ lens consistently maintained better visual acuity across this range, exhibiting a flatter defocus curve and, consequently, a greater depth of focus. ISOPure^®^ demonstrated intermediate performance, whereas Tecnis^®^ Eyhance^™^ showed a more pronounced decline in visual acuity as negative defocus increased. Within the defocus range corresponding to near vision (−2.00 to −3.00 D), AcrySof^®^ IQ Vivity^™^ continued to demonstrate better performance, with consistently better visual acuity values than those achieved with Tecnis^®^ Eyhance^™^ and ISOPure^®^. Both Tecnis^®^ Eyhance^™^ and ISOPure^®^ exhibited a marked deterioration in visual acuity in this range, although ISOPure^®^ tended to show a slight advantage over Tecnis^®^ Eyhance^™^ from −2.00 D onward. When the defocus curves were analyzed using a visual acuity threshold of 0.20 logMAR to define the functional range of vision, clear differences emerged among the three intraocular lenses. The non-diffractive extended depth-of-focus AcrySof^®^ IQ Vivity^™^ provided a functional range of approximately 2.25 diopters toward negative defocus, whereas the enhanced monofocal ISOPure^®^ and Tecnis^®^ Eyhance^™^ lenses achieved narrower ranges of approximately 1.50 diopters and 1.25 diopters, respectively. Importantly, this analysis considered only the myopic defocus range, excluding positive defocus values, in order to reflect clinically meaningful distance-to-near visual performance.

From a clinical perspective, these findings suggest that the non-diffractive extended depth-of-focus AcrySof^®^ IQ Vivity^™^ lens may be particularly suitable for patients with high visual demands at intermediate distances who seek a greater degree of spectacle independence. In contrast, the enhanced monofocal Tecnis^®^ Eyhance^™^ and ISOPure^®^ lenses may be more appropriate for patients who prioritize excellent distance visual quality, accepting the need for additional correction for near tasks.

The defocus profiles observed in the present study are in strong agreement with previously published data. Lesieur et al. [17] reported a similar defocus curve for the ISOPure^®^ lens, characterized by good visual acuity around the optical plane and a gradual decline beyond −1.50 D. Likewise, studies by Bova et al. [14] and Stodulka et al. [18] confirmed the lens’s reliable performance at distance and early intermediate ranges, with limited functional near vision.

For the Tecnis^®^ Eyhance^™^ lens, defocus curves reported by Corbett et al. [19] and Janekova et al. [20] closely match our findings, showing maintained visual acuity up to approximately −1.00 D followed by a steeper decline. These results reinforce the concept that Tecnis^®^ Eyhance^™^ provides a modest extension of intermediate focus compared with conventional monofocal IOLs but does not achieve the broader depth of focus characteristic of EDoF designs such as AcrySof^®^ IQ Vivity^™^. In line with this, Salgado-Borges et al. [21] found no significant differences between ISOPure^®^ and Tecnis^®^ Eyhance^™^ across the defocus curve, with both lenses exhibiting comparable profiles up to −1.50 D, a finding that is fully consistent with the present results.

Regarding AcrySof^®^ IQ Vivity™, the defocus curve profile observed in the present study is consistent with previously published distance-corrected defocus curves of the nondiffractive wavefront-shaping EDoF IOL. Kohnen et al. [22] reported a smooth through-focus behavior with a broad intermediate plateau and a gradual decline toward the near range, closely matching the functional profile observed in our cohort. Similarly, Asena et al. [23] described comparable monocular photopic defocus curves for AcrySof^®^ IQ Vivity™, showing maintained visual acuity across the intermediate defocus range and a progressive reduction beyond −2.00 to −2.50 D. Although absolute logMAR values may vary across studies due to methodological differences, the overall shape and functional behavior of the Vivity defocus curve are consistent.

Contrast sensitivity is a key determinant of functional visual quality, especially in low-light conditions, where small optical differences can have a clinically relevant impact on everyday activities. In the present study, no statistically significant differences in contrast sensitivity were observed between Tecnis^®^ Eyhance^™^, ISOPure^®^, and AcrySof^®^ IQ Vivity^™^ under photopic or mesopic conditions at all spatial frequencies evaluated. This finding is clinically relevant, as one of the traditional limitations of multifocal lenses and some extended depth-of-focus lenses has been reduced contrast sensitivity, typically related to light energy splitting or increased high-order aberrations [9].

Overall, the absence of contrast sensitivity deterioration under mesopic conditions suggests that all three lenses provide a high level of visual quality, suitable for everyday visual tasks, including those performed in low-light environments [9].

Pupil diameter is a well-recognized factor influencing visual performance, particularly under mesopic conditions, as pupil dilation increases the contribution of peripheral optical zones and may accentuate higher-order aberrations [24]. In the present study, mesopic pupil diameter was not significantly correlated with distance or near visual acuity for any of the intraocular lenses evaluated, indicating stable optical performance at these viewing distances despite variations in effective pupil size.

In contrast, a moderate but statistically significant association was observed between mesopic pupil diameter and intermediate visual acuity, with larger pupils being associated with slightly reduced visual acuity. This finding is physiologically plausible and can be explained by the increased influence of spherical aberration and light scatter as the pupil enlarges, effects that tend to have a greater impact on intermediate vision, which relies on a delicate balance between depth of focus and retinal image quality.

From a clinical perspective, these findings indicate that pupil size has a limited influence on distance and near visual outcomes with the lenses evaluated, while intermediate vision may be modestly affected under low-light conditions. Overall, the low pupil dependence observed supports the predictability of visual performance with contemporary enhanced monofocal and non-diffractive extended depth-of-focus intraocular lenses across a broad range of mesopic pupil sizes [25].

Taken together, the clinical outcomes of the three intraocular lenses are consistent with their underlying optical designs. The non-diffractive extended depth-of-focus AcrySof^®^ IQ Vivity™ demonstrated the broadest functional depth of focus, providing better intermediate and near visual performance under both photopic and mesopic conditions and exhibiting a smoother, more extended defocus profile. In contrast, Tecnis^®^ Eyhance™ and ISOPure^®^ showed largely overlapping performance across objective measures, with a modest extension of intermediate vision and a steeper decline at near, supporting their classification as enhanced monofocal rather than true extended depth-of-focus lenses. Importantly, the absence of significant differences in contrast sensitivity or mesopic visual performance suggests that none of the optical strategies evaluated compromise overall visual quality at the early postoperative stage. Collectively, these findings define a coherent functional profile for each IOL category and help clarify the degree of focus extension that can be realistically expected in clinical practice.

Several limitations of this study should be acknowledged. First, the observational design and the absence of random allocation to IOL groups may introduce selection bias related to patient characteristics or surgeon preference. Second, all postoperative evaluations were conducted at a single time point, which does not allow assessment of long-term stability, neuroadaptation phenomena, or potential progressive changes in visual quality. Additionally, analyses were performed monocularly to avoid binocular compensation effects, but this limits the extrapolation to real-world functional vision. The pupil diameter was measured using different instruments under photopic and mesopic conditions, which may introduce inter-device variability despite measurements being obtained under illumination levels matched to those used during visual acuity testing. Furthermore, subjective outcomes, such as patient satisfaction, perception of dysphotopsias, or spectacle Independence, were not assessed, despite their relevance when comparing depth-of-focus–enhancing technologies. Future randomized, longitudinal studies including binocular and patient-reported outcomes would provide a more comprehensive understanding of the comparative performance of these lenses.

## 5. Conclusions

In this prospective comparative study, the non-diffractive extended-depth-of-focus AcrySof^®^ IQ Vivity^™^ IOL provided a significantly wider and more functional range of vision than the two enhanced monofocal lenses evaluated (Tecnis^®^ Eyhance^™^ and ISOPure^®^), with superior intermediate and near visual acuity under both photopic and mesopic conditions. Importantly, this extended depth-of-focus was achieved without a reduction in contrast sensitivity, which remained comparable across all three IOLs.

Tecnis^®^ Eyhance^™^ and ISOPure^®^ demonstrated nearly overlapping performance profiles, offering excellent distance vision and a modest extension of depth-of-focus, but clearly less intermediate and near performance than AcrySof^®^ IQ Vivity^™^.

Further studies with larger cohorts and incorporation of patient-reported outcomes will help refine the clinical selection criteria for these lenses and clarify their relative roles in contemporary cataract surgery.

## Figures and Tables

**Table 1:** Preoperative demographic data for the three lens groups evaluated.

Parameter	Tecnis Eyhance Mean ± SD (Range)	ISOPure Mean ± SD (Range)	AcrySof IQ Vivity Mean ± SD (Range)	*p*-Value
**Age** **(years)**	69.43 ± 6.01 (63/75)	66.88 ± 6.85 (60/74)	65.80 ± 7.90 (58/74)	0.256
**Sex** **(Male/Female; %)**	50/50	50/50	55/45	0.984
**Cataract type (LOCS III)**	NO/NC 1.0–2.5, C/P < 1.0 (40%) NO/NC 2.5–3.5, C/P 2.0–4.0 (50%) NO/NC > 4.0, C/P > 4.0 (10%)	NO/NC 1.0–2.5, C/P < 1.0 (35%) NO/NC 2.5–3.5, C/P 2.0–4.0 (55%) NO/NC > 4.0, C/P > 4.0 (10%)	NO/NC 1.0–2.5, C/P < 1.0 (40%) NO/NC 2.5–3.5, C/P 2.0–4.0 (50%) NO/NC > 4.0, C/P > 4.0 (10%)	
**UDVA** **(logMAR)**	0.56 ± 0.20 (0.20/0.70)	0.49 ± 0.18 (0.16/0.90)	0.60 ± 0.40 (0.24/1.00)	0.360
**CDVA** **(logMAR)**	0.36 ± 0.13 (0.00/0.50)	0.32 ± 0.23 (0.04/0.46)	0.41 ± 0.16 (0.00/0.62)	0.393
**Corneal** **Astigmatism (D)**	0.93 ± 0.25 (0.20/0.99)	0.70 ± 0.28 (0.10/0.98)	0.92 ± 0.24 (0.20/0.99)	0.210
**K1 (D)**	43.67 ± 1.37 (41.40/46.31)	42.99 ± 1.89 (37.78/45.21)	42.98 ± 1.56 (39.90/46.00)	0.382
**K2 (D)**	44.68 ± 1.28 (42.44/47.20)	43.69 ± 1.86 (38.73/46.26)	44.02 ± 1.74 (40.07/46.32)	0.282
**Axial length** **(mm)**	23.63 ± 1.33 (21.21/26.40)	23.91 ± 1.64 (21.47/27.96)	23.54 ± 0.98 (21.52/24.94)	0.988
**IOL power** **(D)**	20.60 ± 3.61 (11.00/27.00)	21.13 ± 3.30 (13.50/27.00)	21.81 ± 2.83 (14.50/27.50)	0.344
**Photopic Pupil size** **(mm)**	2.72 ± 0.45 (2.10/3.60)	2.54 ± 0.61 (1.40/3.40)	2.34 ± 0.47 (1.70/3.40)	0.062
**Mesopic Pupil size** **(mm)**	3.75 ± 0.80 (2.30/5.20)	3.80 ± 1.16 (2.20/6.10)	4.00 ± 0.76 (2.80/5.50)	0.503

UDVA: uncorrected distance visual acuity; CDVA: corrected distance visual acuity; K1, K2: keratometry; mm: millimeters; D: diopters; IOL: intraocular lens; NO/NC: nuclear opalescence/nuclear color; C: Cortical; P: Subcapsular posterior.

**Table 2:** Postoperative manifest refractive outcomes for the three lens groups evaluated.

Postoperative Manifest Refractive Mean ± SD (Range)				
	Tecnis Eyhance (n = 20)	ISOPure (n = 20)	AcrySof IQ Vivity (n = 20)	*p*-Value
**Sphere (D)**	0.07 ± 0.30 (−0.50/1.00)	0.17 ± 0.39 (0.00/1.25)	−0.01 ± 0.10 (−0.25/0.25)	0.119
**Cylinder (D)**	−0.04 ± 0.26 (−0.75/0.00)	−0.23 ± 0.39 (−1.25/0.00)	−0.14 ± 0.39 (−1.00/0.00)	0.166
**Spherical equivalent (D)**	0.01 ± 0.25 (−0.50/0.50)	0.03 ± 0.35 (−0.50/1.00)	−0.07 ± 0.18 (−0.50/0.00)	0.662
**Eyes within ±0.50 D of SE, n (%)**	20/20 (100%)	18/20 (90.0%)	20/20 (100%)	
**Eyes within ±1.00 D of SE, n (%)**	20/20 (100%)	20/20 (100%)	20/20 (100%)	

SE: spherical equivalent; D: diopters.

**Table 3:** Monocular logMAR visual acuity measured at different viewing distances under photopic luminance conditions across the different intraocular lens groups.

Photopic Conditions					
Monocular Visual Acuity (logMAR) Mean ± SD (Range)					
	Tecnis Eyhance (n = 20)	ISOPure (n = 20)	AcrySof IQ Vivity (n = 20)	*p*-Value	Significant Comparisons (*p*-Value)
**UDVA** **(4 m)**	0.00 ± 0.08 (−0.12/0.10)	0.04 ± 0.07 (−0.10/0.14)	−0.04 ± 0.06 (−0.18/0.08)	0.010 *	AcrySof IQ Vivity vs. ISOPure (0.015); AcrySof IQ Vivity vs. Tecnis Eyhance (0.008)
**CDVA** **(4 m)**	−0.06 ± 0.07 (−0.20/0.00)	−0.04 ± 0.06 (−0.16/0.02)	−0.11 ± 0.07 (−0.22/0.00)	0.005 *	AcrySof IQ Vivity vs. ISOPure (0.002)
**DCIVA** **(66 cm)**	0.14 ± 0.06 (0.04/0.22)	0.11 ± 0.07 (0.00/0.20)	0.06 ± 0.06 (0.00/0.20)	0.001 *	AcrySof IQ Vivity vs. ISOPure (0.012); AcrySof IQ Vivity vs. Tecnis Eyhance (<0.001)
**DCNVA** **(40 cm)**	0.50 ± 0.07 (0.32/0.58)	0.45 ± 0.06 (0.32/0.52)	0.32 ± 0.07 (0.20/0.42)	0.015 *	AcrySof IQ Vivity vs. ISOPure (0.002); AcrySof IQ Vivity vs. Tecnis Eyhance (0.003)

UDVA: uncorrected distance visual acuity; CDVA: corrected distance visual acuity; DCIVA: distance-corrected intermediate visual acuity; DCNVA: distance-corrected near visual acuity. * Statistically significant difference *p* < 0.05. Pairwise comparisons (Mann–Whitney U) were considered statistically significant at a Bonferroni-adjusted significance level of α = 0.0167 (3 comparisons).

**Table 4:** Monocular logMAR visual acuity measured at different viewing distances under mesopic luminance conditions across the different intraocular lens groups. Only right eyes were analyzed.

Mesopic Conditions					
Monocular Visual Acuity (logMAR) Mean ± SD (Range)					
	Tecnis Eyhance (n = 20)	ISOPure (n = 20)	AcrySof IQ Vivity (n = 20)	*p*-Value	Significant Comparisons (*p*-Value)
**UDVA** **(4 m)**	0.10 ± 0.09 (0.00/0.22)	0.06 ± 0.05 (−0.02/0.30)	0.00 ± 0.07 (−0.12/0.20)	0.145	—
**CDVA** **(4 m)**	0.09 ± 0.08 (−0.10/0.18)	0.07 ± 0.06 (−0.08/0.24)	0.04 ± 0.11 (−0.22/0.16)	0.231	—
**DCIVA** **(66 cm)**	0.41 ± 0.11 (0.30/0.68)	0.46 ± 0.12 (0.30/0.66)	0.35 ± 0.08 (0.20/0.52)	0.007 *	AcrySof IQ Vivity vs. ISOPure (0.002)
**DCNVA** **(40 cm)**	0.68 ± 0.05 (0.54/0.74)	0.71 ± 0.07 (0.56/0.80)	0.58 ± 0.10 (0.40/0.72)	0.043 *	AcrySof IQ Vivity vs. ISOPure (0.013); AcrySof IQ Vivity vs. Tecnis Eyhance (0.006)

UDVA: uncorrected distance visual acuity; CDVA: corrected distance visual acuity. DCIVA: distance-corrected intermediate visual acuity; DCNVA: distance-corrected near visual acuity; m: meter; mm: millimeter. * Statistically significant difference *p* < 0.05. Pairwise comparisons (Mann–Whitney U) were considered statistically significant at a Bonferroni-adjusted significance level of α = 0.0167 (3 comparisons).

**Table 5:** Monocular contrast sensitivity (logCS) under photopic and mesopic conditions in the different intraocular lens groups. Analysis was limited to right eyes.

Contrast Sensitivity				
Photopic Conditions Mean ± SD (Range)				
	Tecnis Eyhance (n = 20)	ISOPure (n = 20)	AcrySof IQ Vivity (n = 20)	*p*-Value
**3 cpd**	1.92 ± 0.09 (1.78/2.08)	1.90 ± 0.13 (1.49/2.08)	1.95 ± 0.13 (1.78/2.08)	0.433
**6 cpd**	2.14 ± 0.11 (1.99/2.29)	2.10 ± 0.16 (1.70/2.29)	2.15 ± 0.06 (1.99/2.29)	0.821
**12 cpd**	1.85 ± 0.13 (1.69/1.99)	1.80 ± 0.21 (1.25/1.99)	1.83 ± 0.13 (1.54/1.99)	0.861
**18 cpd**	1.44 ± 0.12 (1.25/1.55)	1.35 ± 0.22 (0.96/1.55)	1.43 ± 0.13 (1.10/1.55)	0.184
**Mesopic Conditions** **Mean ± SD (Range)**				
	**Tecnis Eyhance** **(n = 20)**	**ISOPure** **(n = 20)**	**AcrySof IQ Vivity** **(n = 20)**	***p*** **-Value**
**3 cpd**	1.84 ± 0.08 (1.78/1.93)	1.86 ± 0.18 (1.34/2.08)	1.86 ± 0.11 (1.63/2.08)	0.659
**6 cpd**	2.05 ± 0.10 (1.84/2.14)	2.01 ± 0.21 (1.55/2.29)	2.07 ± 0.09 (1.84/2.14)	0.967
**12 cpd**	1.77 ± 0.13 (1.54/1.99)	1.77 ± 0.22 (1.25/1.99)	1.79 ± 0.20 (1.40/1.99)	0.670
**18 cpd**	1.37 ± 0.15 (0.96/1.55)	1.25 ± 0.19 (0.81/1.55)	1.35 ± 0.15 (0.96/1.55)	0.051

cpd: cycles per degree.

## Data Availability

The data presented in this study are not publicly available due to patient privacy and ethical restrictions. Data may be made available upon reasonable request to the corresponding author, subject to approval by the institutional ethics committee.

## Appendix A

**Table A1:** Global tests from the linear mixed-effects model for the monocular defocus curve (logMAR) under photopic conditions.

Effect	LR Chi-Square	df	*p*-Value
IOL × Defocus interaction	857.59	16	<0.001
IOL (main effect)	1003.38	18	<0.001
Defocus (main effect)	2423.02	24	<0.001

IOL: intraocular lens; LR χ^2^: likelihood-ratio chi-square statistic; df: degrees of freedom; logMAR: logarithm of the minimum angle of resolution.
